# In this month’s Bulletin

**DOI:** 10.2471/BLT.20.000320

**Published:** 2020-03-01

In the editorial section, Vasee Moorthy et al. (150) announce a WHO pre-print server for research on the novel coronavirus (2019-nCoV). Peter Beyer & Sarah Paulin (151) provide an update on priority pathogens and the antibiotic pipeline. Payao Phonsuk et al. (152) announce an upcoming theme issue and call for papers on the health impacts of climate change and geopolitics. Darren B. Taichman et al. (153) propose a new disclosure form for the authors submitting work to medical journals.

In the news section, Lynne Eaton (156–157) reports on global supply chain problems with heparin and magnesium sulfate. Karla Soares-Weiser (158–159) talks to Gary Humphreys about systematic reviews of evidence and the Cochrane Library.

## Bangladesh, Benin, Democratic Republic of the Congo, Egypt, Guyana, Haiti, Kenya, Malawi, Mauritania, Namibia, Nepal, Rwanda, Senegal, Sierra Leone, Somalia, Togo, Uganda, United Republic of Tanzania, Zambia, Zimbabwe

### Reliable access to antibiotics

Rebecca Knowles et al. (176–188) collate data from 20 low- and middle-income countries.

## Colombia

### What works to reduce fatal shootings?

Andres I Vecino-Ortiz & Deivis N Guzman-Tordecilla (169–175) study gun-carrying restrictions and gun-related mortality

## India

### Medical devices and adverse events

Shatrunajay Shukla et al. (207–212) describe the expansion of regulatory scope to medical devices. 

## Kazakhstan

### Treating hypertension, diabetes and chronic heart failure

Benjamin TB Chan et al. (160–168) evaluate measures to improve quality of care for patients with chronic diseases. 

## Thailand

### Preventing tuberculosis transmission

Worarat Imsanguan et al. (83–84) provide infection prevention recommendations for contacts of people with tuberculosis.

## Global

### Treating hepatitis C 

Hugo Perazzo et al. (189–198) review the evidence of effectiveness for generic direct-acting agents.

### Platyhelminth control

Matthew A Dixon et al. (199–206) model the effects of *Taenia solium* control strategies.

### Hidden determinants of early childhood outcomes

Gautam Bhan (220–222) examine the links between informal work and maternal and child health.

### Reducing the harmful use of alcohol

David H Jernigan & Pamela J Trangenstein (223–225) explore next steps for WHO’s global alcohol strategy.

### Factors that influence physical activity 

James L Nuzzo & James Steele (226–227) argue for a causal systems map.

Harry Rutter et al. (227–228) outline systems approaches to support physical activity.

